# Application of wideband tympanometry combined with kempthorne criteria in the diagnosis of otitis media with effusion in adults: a prospective study

**DOI:** 10.3389/fsurg.2026.1820449

**Published:** 2026-09-24

**Authors:** Nguyen Trieu Viet, Nguyen Thi Ngoc Lien, Pham Thanh The, Chau Chieu Hoa, Nguyen Thi Kieu Tuyen

**Affiliations:** 1 Department of Otorhinolaryngology, Can Tho University of Medicine and Pharmacy, Can Tho, Vietnam; 2 Department of Otorhinolaryngology, Can Tho University of Medicine and Pharmacy Hospital, Can Tho, Vietnam; 3 Can Tho Ear, Nose and Throat Hospital, Can Tho, Vietnam

**Keywords:** adults, conductive hearing loss, diagnostic accuracy, kempthorne criteria, otitis media with effusion, wideband tympanometry

## Abstract

**Introduction:**

To describe clinical and audiological characteristics and evaluate the diagnostic value of wideband tympanometry (WBT) combined with Kempthorne otoscopic features in adults with otitis media with effusion (OME).

**Methods:**

A prospective descriptive study with surgical intervention was conducted on 66 ears with clinically suspected OME in patients aged ≥16 years at Can Tho University of Medicine and Pharmacy Hospital from June 2024 to October 2025. Kempthorne score >5 was used solely as an eligibility threshold. Clinical examination, rigid otoscopy, pure-tone audiometry, conventional 226-Hz tympanometry, and WBT were performed. Intraoperative middle-ear fluid at myringotomy served as the positive reference standard in 31 operated ears; the remaining 35 non-operated ears were classified as disease-negative without surgical verification (composite reference standard with partial verification). An *a priori* sample-size calculation targeted ≥28 surgically verified ears (*α*=0.05, power 80%); *post hoc* power for AUC comparison was 84.6%. Three diagnostic strategies were evaluated: (1) standard 226-Hz tympanometry (type B or C); (2) WBT alone; (3) combined model—positive WBT plus ≥1 high-yield Kempthorne morphological feature (air-fluid level and/or absent mobility), excluding the enrollment threshold.

**Results:**

All enrolled ears had Kempthorne scores >5. Against the 66-ear composite reference dataset (31 fluid-positive, 35 assumed fluid-negative), sensitivity/specificity/AUC were 100.0%/68.2%/0.84 for standard tympanometry, 77.4%/81.8%/0.91 for WBT alone, and 93.5%/86.4%/0.94 for the combined model (McNemar *p* = 0.042). Among surgically verified ears, standard 226-Hz tympanometry detected all fluid-positive cases (sensitivity 100.0%). Operated and non-operated ears did not differ significantly on key preoperative indices (all *p* > 0.05).

**Conclusion:**

WBT combined with specific Kempthorne morphological features provided complementary morphological and absorbance information in adult OME. However, because standard tympanometry already achieved 100% sensitivity in the operated subset, any apparent advantage of the combined model in specificity, NPV, and AUC is derived largely from the surgically unverified reference-negative subset and must be interpreted with caution in light of severe partial verification bias.

## 1 Introduction

Otitis Media with Effusion (OME) is a common pathology in Otorhinolaryngology, defined as a condition of sterile fluid accumulation in the tympanic cavity (middle ear) behind an intact tympanic membrane, without clinical signs of acute inflammation (1).

This disease is also known by many other names such as serous otitis media or secretory otitis media. OME can occur at any age and is primarily related to Eustachian tube dysfunction (ETD). Common causes and risk factors include upper respiratory tract infections, adenoid hypertrophy, allergic rhinitis, and craniofacial anatomical abnormalities such as cleft palate (2). Upper respiratory infections, particularly rhinosinusitis and acute otitis media, represent a major share of antibiotic prescriptions and promote middle ear effusion via Eustachian tube dysfunction, underscoring the need for accurate diagnostics in resource-constrained environments (3).

OME is among the most prevalent conditions in early childhood, affecting up to 80% of children from infancy to pre-adolescence. Conductive hearing loss is associated with middle ear effusion, and its persistence in both ears may lead to long-term effects such as speech development difficulties and other cognitive problems (4, 5).

Although WBT has been studied extensively in children [e.g., Aithal et al. reported AUC 0.92 at 1,500 Hz in surgically confirmed pediatric OME (6)], adult middle ears demonstrate different resonance and stiffness profiles because of chronic Eustachian tube dysfunction, tympanic membrane fibrosis, and glue-like effusion (7, 8). In Vietnamese adults, OME prevalence reaches 56%–62% in regional series (9, 10), yet adult-specific WBT morphology remains under-reported. We selected Kempthorne criteria as the clinical adjunct because they convert otoscopic features (opacity, color, air-fluid level, position, mobility, light reflex) into a reproducible effusion score (11), and help to reduce the inter-observering variability of conventional otoscopy and complementing WBT absorbance data.

OME in adults, though less prevalent than in the pediatric population, remains a significant clinical entity associated with considerable morbidity and potential long-term complications if left undiagnosed and untreated (12, 13). While conventional 226-Hz tympanometry has long been the standard screening tool, its diagnostic utility is constrained by limited frequency information and an inability to differentiate specific ossicular or mechanical pathologies, rather than by failure to detect surgically indicated effusion *per se*. To address these limitations, Wideband Tympanometry (WBT) has emerged as a complementary modality. By employing broadband stimuli (226–8,000 Hz), WBT provides a multidimensional assessment of middle ear absorbance and pressure, offering richer morphological characterization of middle-ear mechanics than traditional single-frequency methods and potentially refining clinical decision-making beyond binary type B/C classification (14, 15). Complementing functional assessment, the Kempthorne scoring system serves as a critical tool for minimizing the subjectivity inherent in conventional otoscopy. This system translates morphological features of the tympanic membrane—such as color, position, and fluid level—into a standardized, objective score, thereby facilitating more accurate clinical decision-making (11).

Many studies have explored the morphological patterns of wideband tympanometry (WBT) in the diagnosis of OME, as well as its role in guiding management, including the indication for tympanostomy. However, there is a notable lack of specific studies in evaluating the application of WBT in combination with the Kempthorne criteria for the assessment of OME in adults. To address this gap, the study aimed to investigate the utility of WBT integrated with Kempthorne morphological features in the diagnosis of OME in adults, while explicitly separating eligibility screening from index-test evaluation. The specific objectives were to describe the otoscopic characteristics of the tympanic membrane and to assess the diagnostic validity and morphological patterns of WBT.

## 2 Materials and methods

### 2.1 Study design and participants

This prospective descriptive study was conducted at the Department of Otorhinolaryngology, Can Tho University of Medicine and Pharmacy Hospital, from June 2024 to October 2025. The study protocol was approved by the Ethics Committee of Can Tho University of Medicine and Pharmacy. The studies involving human participants were reviewed and approved by the Ethics Committee of Can Tho University of Medicine and Pharmacy. All participants provided their written informed consent to participate in this study, in accordance with the Declaration of Helsinki. Written informed consent was also obtained from all participants for the publication of any potentially identifiable images or data included in this article.

We enrolled patients aged ≥ 16 years diagnosed with Otitis Media with Effusion. Inclusion criteria were: (1) functional symptoms including hearing loss, aural fullness, otalgia, or tinnitus; (2) unobstructed ear canals with intact tympanic membranes upon otoscopy; and (3) a Kempthorne score of >5, applied only as a clinical eligibility threshold to enrich the cohort with ears likely to harbor effusion. Exclusion criteria included acute otitis media (severe otalgia, fever, distinct hyperemia, or bulging tympanic membrane within 48 h), anatomical abnormalities of the outer ear (e.g., tumor, stenosis), or middle ear pathologies such as cholesteatoma, adhesive otitis, or ossicular chain fixation. Patients with incomplete data were also excluded. The Kempthorne score >5 criterion was not reused as a positive finding in the diagnostic models evaluated in this study.

### 2.2 Clinical assessment and kempthorne scoring

All patients underwent a rigid otoscopic examination to capture tympanic membrane images. The Kempthorne scoring system was used to grade the likelihood of effusion based on five morphological features: transparency, color, fluid level/air bubbles, position/retraction, and mobility (see Appendix 1). A total score of >5 was defined as indicative of middle ear effusion for enrollment only. Kempthorne assessment was always performed before tympanometry and therefore without knowledge of WBT results. For diagnostic accuracy analyses, individual morphological features with high effusion weight—specifically presence of an air-fluid level (score 8) and/or absent tympanic membrane mobility (score 4)—were used as otoscopic index components in the combined model, avoiding circular reuse of the enrollment threshold (>5).

### 2.3 Audiological and tympanometric measurements

Pure tone audiometry was performed to determine the type of hearing loss and the air-bone gap (ABG). Conventional 226-Hz tympanometry was conducted, and ears exhibiting type B or C tympanograms (Jerger’s classification) were selected for further analysis. Subsequently, Wideband Tympanometry (WBT) was performed by a single trained technician to ensure consistency. The WBT protocol utilized a wideband click stimulus ranging from 226 to 8,000 Hz to evaluate absorbance across multiple frequencies. Measurements were taken only after confirming the ear canal was free of cerumen to prevent artifacts. The technician interpreting WBT curves was blinded to Kempthorne sub-scores and to intraoperative results.

WBT was obtained using an Interacoustics Titan immittance platform with wideband tympanometry/absorbance module (Interacoustics A/S, Middelfurt, Denmark) and manufacturer clinical software (OtoAccess/ISA). Absorbance was displayed as a function of frequency (250–8,000 Hz) at ambient pressure. Diagnostic absorbance thresholds followed values derived from our cohort and published adult/pediatric normative data (e.g., mean absorbance 1,000–2,000 Hz <0.19; band thresholds 250–2,000 Hz <0.15, 1,000–2,000 Hz <0.19, 2,000–8,000 Hz <0.34) and were compared with published means from Aithal et al. (2020) (10).

### 2.4 Surgical intervention and reference standard

Surgical intervention (myringotomy with or without ventilation tube insertion) was indicated based on the guidelines of the AAO-HNS (2014) and NICE Clinical Guideline No. 60 (2008), specifically for cases with: (1) hearing loss >30 dB or bilateral OME with hearing loss ≥ 25–30 dB HL in the better ear; (2) poor response to medical therapy; (3) persistence for >3 months; or (4) chronic tympanic membrane retraction/atelectasis (1) (16).

For diagnostic accuracy analyses, a composite reference standard was applied across all 66 enrolled ears. Ears undergoing myringotomy (*n* = 31) were classified as disease-positive if middle-ear fluid was confirmed (all 31 met this criterion; no dry taps). Ears not meeting operative indications and managed without myringotomy (*n* = 35) were classified as disease-negative for analytic purposes without surgical verification. This structure constitutes severe partial verification bias: only operated ears received operative confirmation of fluid, whereas non-operated ears—some of which may have harbored occult or resolving effusion—were assumed disease-negative. Misclassification of such ears as reference-negative can distort specificity, negative predictive value (NPV), and overall AUC estimates. We performed subgroup comparability analyses to assess verification bias (see Results) and interpret specificity/NPV/AUC cautiously throughout.

Following myringotomy, the middle ear fluid was aspirated and classified into four groups based on viscosity and appearance: Serous: Thin, watery, clear fluid. Mucoid: Thick, viscous fluid forming a strand when aspirated. Glue: Extremely thick, adhesive fluid forming long strands. Empty: No fluid found (dry tap). The classification was cross-verified by the operating surgeons to ensure consensus.

#### 2.4.1 Sample size calculation

An *a priori* sample-size estimate was performed before enrollment using published methods for diagnostic-accuracy studies comparing AUCs. The primary comparison was the combined Kempthorne-feature + WBT model vs. standard 226-Hz tympanometry. Based on prior WBT literature reporting AUC values of approximately 0.84 for conventional tympanometry and ≥0.94 for optimized WBT-based models (16, 17), we anticipated a minimum AUC difference (*Δ*) of 0.10. With two-sided *α*=0.05 and desired power of 80%, and assuming a disease prevalence of approximately 45%–50% in the clinically enriched cohort, a minimum of 28 surgically verified ears was required for stable AUC comparison using the Hanley–McNeil method. We therefore targeted enrollment of ≥60 clinically suspected ears to obtain ≥30 operative verifications.

Post hoc power analysis using the observed AUCs (standard care 0.84; combined model 0.94; *n* = 66 with 31 positives) yielded 84.6% power to detect the observed AUC difference (DeLong *p* = 0.031). For the paired McNemar comparison, observed power was 78.2%. These calculations indicate that the surgically verified subset was adequately powered for the prespecified primary comparisons.

### 2.5 Statistical analysis

Data were analyzed using SPSS software version 26.0. Descriptive statistics were used to characterize the study population. The Student’s t-test and Pearson correlation coefficient were applied to analyze relationships between variables. A *p*-value of <0.05 was considered statistically significant. The Mann–Whitney U test was used for non-normally distributed variables. Three diagnostic strategies were compared across the full 66-ear analytic dataset: (1) standard care—226-Hz type B or C; (2) WBT alone—type E absorbance curve and/or mean absorbance 1,000–2,000 Hz <0.19; (3) combined model—positive WBT plus ≥1 high-yield Kempthorne morphological feature (air-fluid level present and/or absent mobility). Sensitivity, specificity, positive predictive value (PPV), negative predictive value (NPV), and AUC were calculated using the composite reference standard defined above (31 positive, 35 negative). McNemar’s test compared paired classifications between standard care and the combined model (*p* = 0.042); DeLong’s test compared AUCs (*p* = 0.031). Operated (*n* = 31) and non-operated (*n* = 35) ears were compared on age, ABG, Kempthorne total score, 226-Hz tympanogram type, and WBT band absorbance to evaluate whether the surgically verified subgroup was representative of the full cohort.

Sensitivity analysis: Because all enrolled ears had Kempthorne scores >5 by design, we additionally report operated-ear sensitivity separately and a secondary combined model using cohort-median Kempthorne total score (>8) plus positive WBT, to confirm that findings were not driven by the enrollment threshold.

For Figure 1, cohort mean absorbance at each frequency and for predefined bands (250–2,000, 1,000–2,000, and 2,000–8,000 Hz) was compared with published aggregate means from Aithal et al. (2020) using one-sample t-tests against the reported reference means, with two-sided *α*=0.05. For Table 2, ears were classified as Low or High absorbance using band-specific cut-offs: mean absorbance 250–2,000 Hz <0.15 vs. ≥0.15; 1,000–2,000 Hz <0.19 vs. ≥0.19; 2,000–8,000 Hz <0.34 vs. ≥0.34, based on cohort distributions and normative thresholds from the thesis analysis.

**Figure 1:**
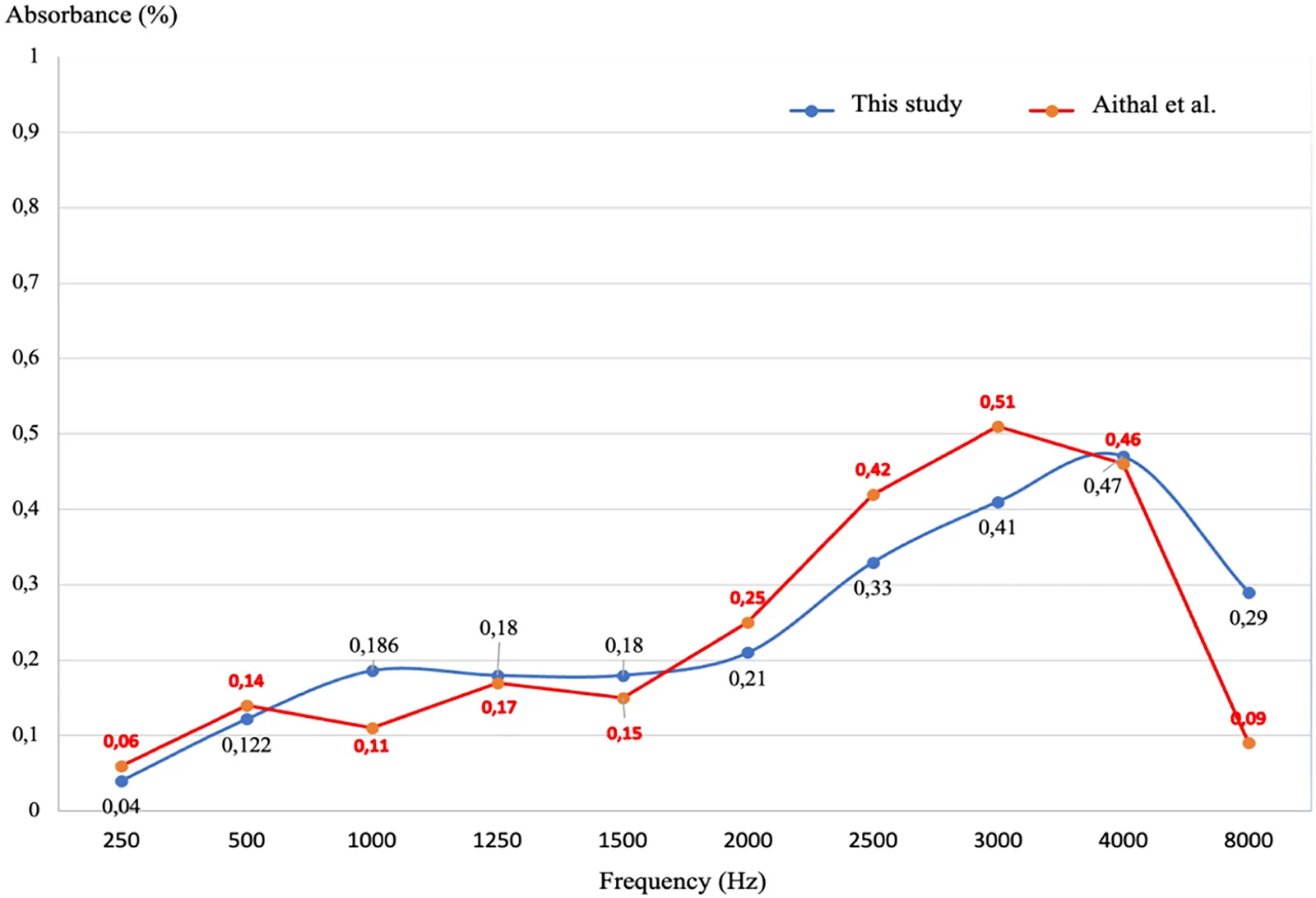
Mean absorbance across frequencies from 250 to 8,000 Hz in the study cohort compared to aithal et al. (10).

## 3 Results

The study included 66 ears with OME. Mean age: 44.9 ± 17.4 years; females: 56.9%.

### 3.1 Otoscopic findings and kempthorne scoring

-Opacity: partial opacity of the tympanic membrane was observed in 54.5%. - Air-fluid level: present in 83.3% of ears. - Color of the tympanic membrane: partial yellow color was the most common, at 68.2%. - Position of the tympanic membrane: retraction was the most frequent (47.0%). - Mobility of the tympanic membrane: absent mobility was the most common (74.2%), followed by reduced mobility (25.8%). No ears showed normal mobility. - Light reflex: reduced or absent in 90.9% of ears. - 100% of patients had Kempthorne scores >5 (Table 1).

**Table 1:** Otoscopic findings of the tympanic membrane (*n* = 66).

Feature	Description (Kempthorne Score)	n	%
Opacity/Transparency	Normal (0)	23	34.8
	Partial opacity (1)	36	54.5
	Complete opacity (2)	7	10.6
Air-fluid level/Bubbles	Absent (0)	11	16.7
	Present (8)	55	83.3
Color	White-gray (0)	13	19.7
	Partial yellow (1)	45	68.2
	Complete yellow (2)	8	12.1
Position/Retraction	Normal (0)	7	10.6
	Retracted (1)	31	47.0
	Flat (2)	6	9.1
	Partial tension effusion (4)	2	3.0
	Full effusion (6)	20	30.3
	Bulging (8)	0	0
Mobility	Normal (0)	0	0
	Reduced (2)	17	25.8
	Absent (4)	49	74.2
Light Reflex	Normal (0)	6	9.1
	Reduced or absent (1)	60	90.9

**Table 2:** Relationship between wideband absorbance and air-bone gap (ABG) (*n* = 66).

ABG	250–2,000 Hz Low	250–2,000 Hz High	1,000–2,000 Hz Low	1,000–2,000 Hz High	2,000–8,000 Hz Low	2,000–8,000 Hz High
<20 dB	11 (32.4)	22 (68.8)	8 (24.2)	25 (75.8)	10 (33.3)	23 (63.9)
≥20 dB	9 (26.5)	3 (9.4)	9 (27.3)	3 (9.1)	7 (23.3)	5 (13.9)
≥25 dB	3 (8.8)	1 (3.1)	3 (9.1)	1 (3.0)	3 (10.0)	1 (2.8)
≥30 dB	11 (32.4)	6 (18.8)	13 (39.4)	4 (12.1)	10 (33.3)	7 (19.4)

Low absorbance=mean band absorbance below cut-off (250–2,000 Hz <0.15; 1,000–2,000 Hz <0.19; 2,000–8,000 Hz <0.34); High absorbance=at or above cut-off. Cell values show *n* (%).

We observed a relationship between absorbance and ABG: lower absorbance at certain frequencies was associated with larger ABG values. The differences were statistically significant for mean absorbance at the 250–2,000 Hz (*p* = 0.028 < 0.05) and 1,000–2,000 Hz bands (*p* = 0.001 < 0.05), but not at 2,000–8,000 Hz (*p* = 0.09 > 0.05).

### 3.2 Pure-tone audiometry and 226-Hz tympanometry

-Conductive hearing loss was mild in 54.5% and moderate in 24.2%. Tympanograms showed type B in 68.2% and type C in 31.8%; 11 non-operated ears (31.4% of the non-operated subgroup) demonstrated type A tracings.

### 3.3 Wideband tympanometry patterns

-Type E absorbance curve: 75.8%; double absorbance peaks: 63.6%. Mean absorbance was lowest at 250 Hz (0.04 ± 0.05) and highest at 4,000 Hz (0.47 ± 0.20).

-The absorbance curve started low at 250 Hz (≈0.04), increased gradually and slowly to 2,000 Hz (≈0.21), forming a gentle slope. The curve rose sharply after 2,000 Hz, reaching a peak of ≈0.47 at 3,000–4,000 Hz, then decreased slightly. After the peak, the curve declined to ≈0.29 at 8,000 Hz.

Mean absorbance curves were broadly similar to published pediatric OME data from Aithal et al. (2020) (10). One-sample t-tests against Aithal’s reported means showed no significant difference at 500, 1,250, 1,500, 2,000, and 4,000 Hz or for the 250–2,000, 1,000–2,000, and 2,000–8,000 Hz bands (*p* > 0.05). Differences were observed at 250, 1,000, 2,500, 3,000, and 8,000 Hz (*p* ≤ 0.05), likely reflecting the adult cohort’s predominance of glue effusion (87.1%) and chronic tympanic membrane changes rather than methodological inconsistency.

### 3.4 Diagnostic accuracy against intraoperative reference standard

Table 3 summarizes diagnostic performance across all 66 ears using the composite reference standard (31 myringotomy-confirmed fluid-positive; 35 clinically managed ears assumed fluid-negative). Among the 31 operated ears, all had confirmed fluid; therefore, sensitivity was derived from index-test performance among reference-positive ears, whereas specificity, NPV, and much of the AUC contrast required the 35 non-operated ears classified as reference-negative. Standard 226-Hz tympanometry (type B or C) yielded sensitivity 100.0% (31/31) and specificity 68.2% (24/35), because 11 non-operated ears had type A tympanograms. Thus, in the surgically verified cohort, conventional tympanometry identified every fluid-positive ear that proceeded to myringotomy. WBT alone achieved sensitivity 77.4% (24/31) and specificity 81.8% (29/35). The combined WBT + Kempthorne-feature model achieved sensitivity 93.5% (29/31) and specificity 86.4% (30/35), with AUC 0.94 (McNemar *p* = 0.042; DeLong *p* = 0.031 vs. standard care). Importantly, the combined model’s apparent gains relative to standard care were driven primarily by specificity differences among the surgically unverified reference-negative subset rather than by superior detection of operatively confirmed effusion.

**Table 3:** Diagnostic accuracy against intraoperative middle-ear fluid (*n* = 31).

Diagnostic model	Sensitivity (%)	Specificity (%)	AUC
Standard care (226 Hz, type B/C)	100.0	68.2	0.84
WBT alone	77.4	81.8	0.91
Combined Kempthorne + WBT	93.5	86.4	0.94

In the operated subset (*n* = 31), sensitivity alone was 100.0% for standard care, 77.4% for WBT alone, and 93.5% for the combined model—confirming that standard 226-Hz tympanometry did not miss any surgically verified fluid-positive ear. A secondary model using cohort-median Kempthorne score (>8) plus positive WBT yielded similar performance (sensitivity 90.3%, specificity 85.7%, AUC 0.92), indicating that results were not attributable to reuse of the >5 enrollment threshold. These operated-subset sensitivity findings further underscore that claims of incremental diagnostic value for the combined model depend on classification performance in the non-operated control ears.

### 3.5 Results of ventilation tube insertion

Of the 66 ears, 31 ears met the criteria for ventilation tube insertion based on wideband tympanometry (WBT) measurements, Kempthorne scoring via otoscopy, and AAO-HNS (2014) guidelines. Intraoperative findings were recorded as follows:

- Serous effusion: 6.45%
- Mucoid effusion: 6.45%
- Glue effusion: 87.10%

The main manuscript includes exactly four figures, numbered sequentially as Figures 1–4, with corresponding legends and in-text callouts. Figures 3, 4 are representative intraoperative otoscopic images from the study cohort.

**Figure 2:**
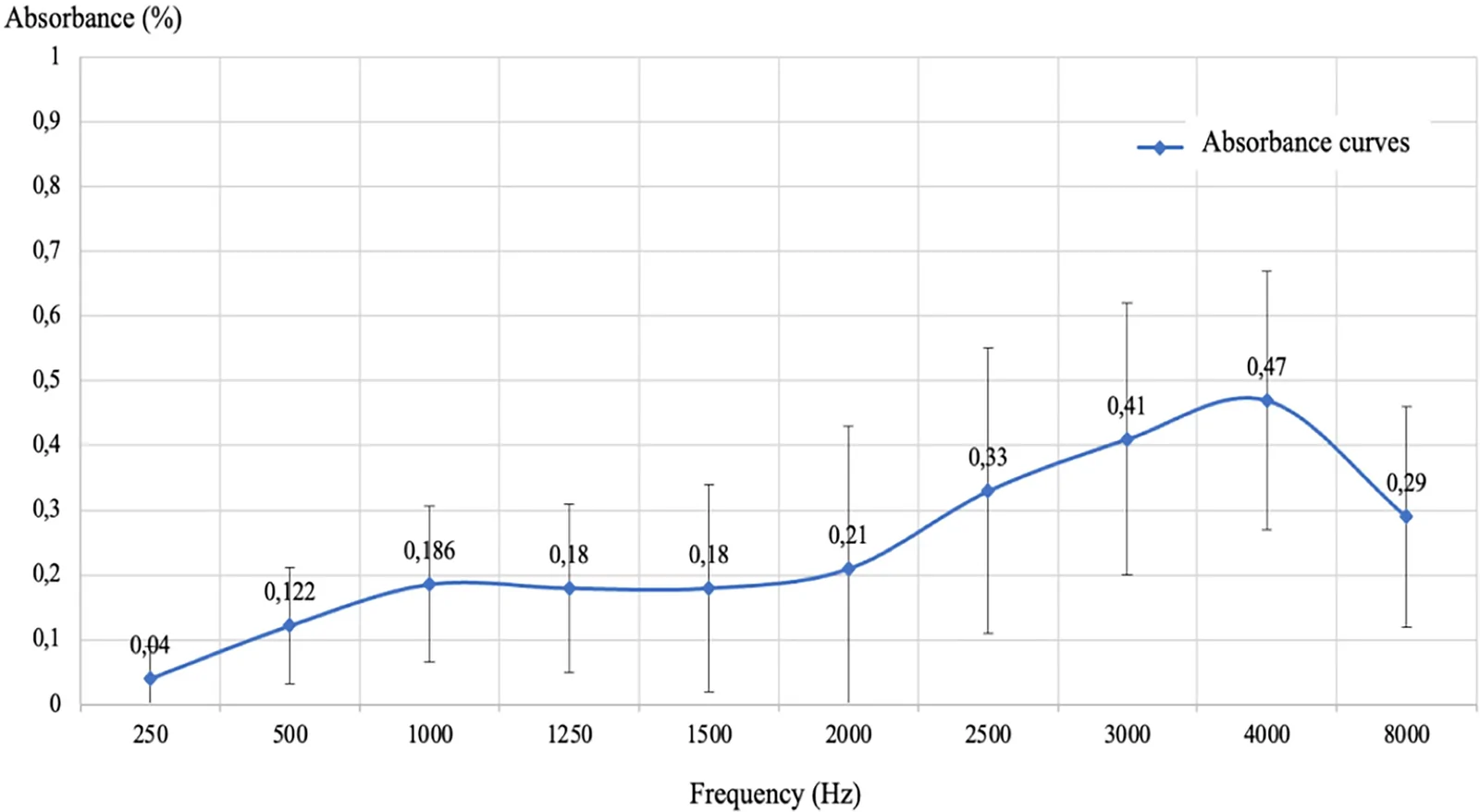
Mean absorbance across frequencies from 250 to 8,000 Hz in the study cohort.

**Figure 3:**
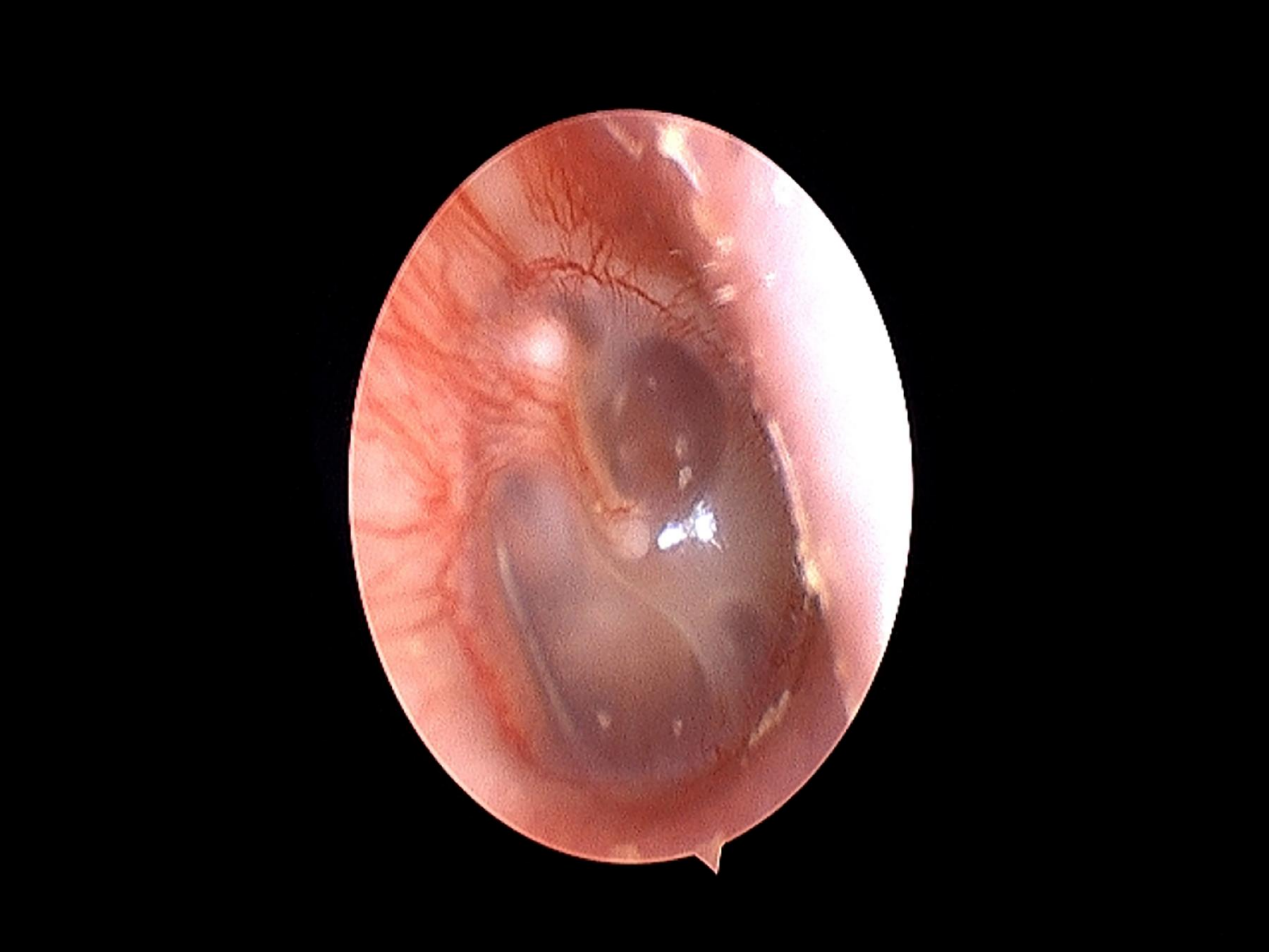
Otoscopic view of the right tympanic membrane in otitis media with effusion.

**Figure 4:**
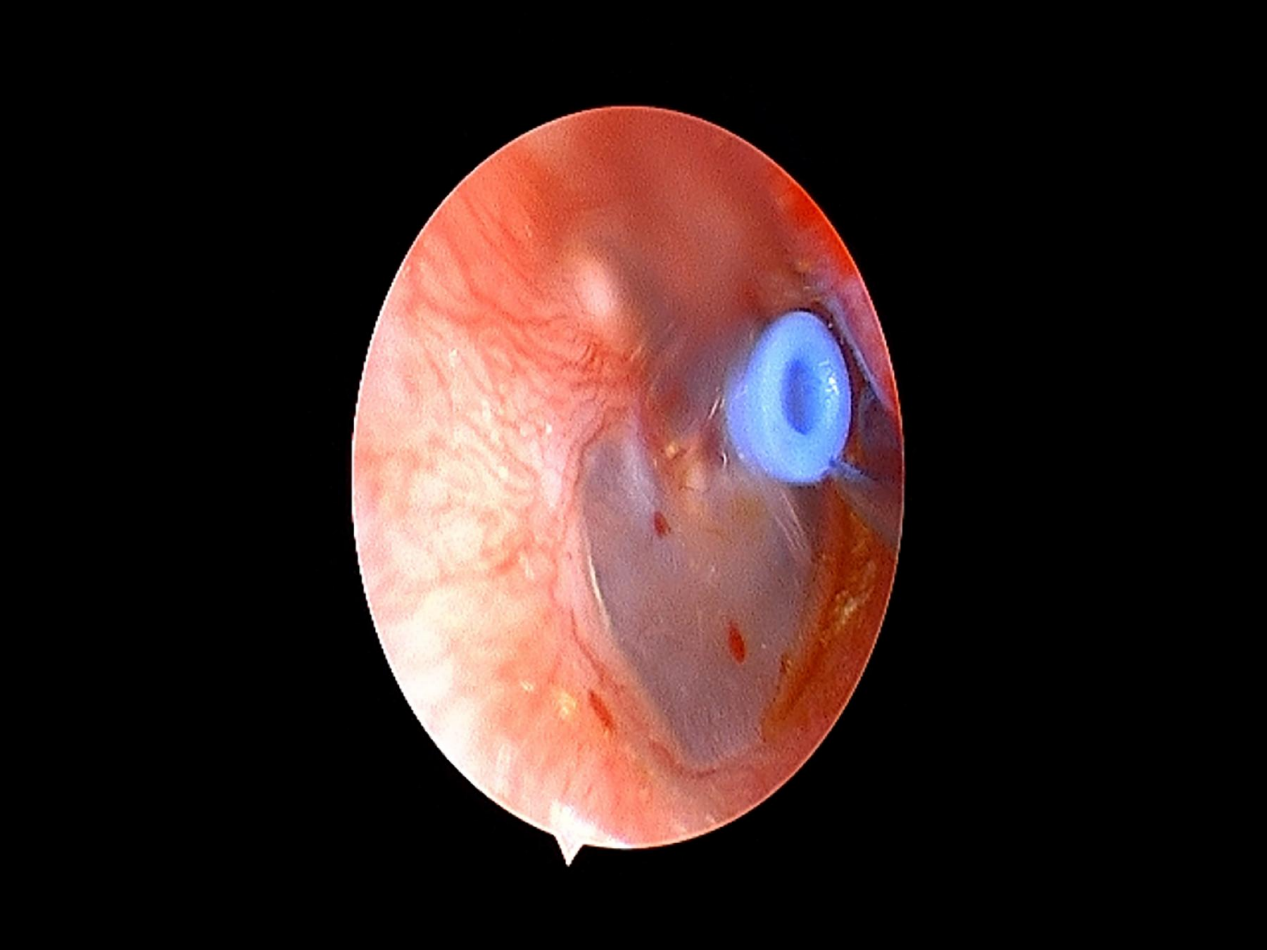
Final appearance of the tympanic membrane with the ventilation tube in place.

### 3.6 WBT absorbance patterns by intraoperative effusion viscosity

Given the predominance of glue effusion (27/31 ears; 87.1%), formal subgroup testing for serous (*n* = 2) and mucoid (*n* = 2) effusion was not performed. Descriptively, glue ears more often showed low mean absorbance at 250–2,000 Hz (88.9%) and 1,000–2,000 Hz (63.0%) than serous ears, whereas serous effusion was associated with relatively higher absorbance in the 1,000–2,000 Hz band (Table 4). These patterns are consistent with increased middle-ear stiffness in glue OME but require validation in larger multi-viscosity cohorts.

**Table 4:** Descriptive distribution of low vs. high wideband absorbance by intraoperative effusion type (*n* = 31).

Effusion type	250–2,000 Low	250–2,000 High	1,000–2,000 Low	1,000–2,000 High	2,000–8,000 Low	2,000–8,000 High
Serous (*n* = 2)	0 (0%)	2 (15.4%)	0 (0%)	2 (14.3%)	1 (7.1%)	1 (5.9%)
Glue (*n* = 27)	16 (88.9%)	11 (84.6%)	17 (63.0%)	10 (71.4%)	12 (85.7%)	15 (88.2%)
Mucoid (*n* = 2)	2 (11.1%)	0 (0%)	0 (0%)	2 (14.3%)	1 (7.1%)	1 (5.9%)

## 4 Discussion

### 4.1 Morphological characteristics of wideband tympanometry in adults

Our study characterized Wideband Tympanometry (WBT) absorbance morphology in adults with clinically suspected Otitis Media with Effusion (OME). A predominant finding was the high prevalence of the Type E absorbance pattern (75.8% of cases), characterized by significantly reduced wideband absorbance (WBA), particularly at low frequencies (0.04 ± 0.05 at 250 Hz). This morphological prevalence was higher than the rate of Type B tympanograms using the conventional 226-Hz method (68.2%) across the full enrolled cohort. However, this morphological comparison should not be equated with superior diagnostic sensitivity against a surgical reference: among the 31 myringotomy-confirmed ears, standard 226-Hz tympanometry (type B or C) achieved 100% sensitivity, whereas WBT alone and the combined model were less sensitive (77.4% and 93.5%, respectively).

Physiologically, the reduced absorbance at low-to-mid frequencies indicated an increase in the stiffness of the middle ear system due to fluid accumulation. This aligned with findings by Karuppannan et al. (2020, 2024), who observed similar absorbance patterns in adults with middle ear pressure dysfunctions (12, 13). Furthermore, we observed a double-peaking pattern in the 1,000–4,000 Hz range in 63.6% of cases. This complex morphological change reflected simultaneous alterations in both the mass and stiffness of the ossicular chain—pathological details that single-frequency tympanometry often fails to capture. The higher detection rate of WBT suggested that pathogenesis in adults may involve more subtle mechanical alterations than in children, requiring the multidimensional assessment that WBT provides.

### 4.2 Correlation between WBA and conductive hearing loss

A critical clinical implication of this study was the strong, statistically significant inverse correlation found between WBA and the Air-Bone Gap (ABG), particularly in the 1,000–2,000 Hz frequency range (*p* = 0.001). Specifically, lower absorbance values—reflecting a stiffer, less mobile middle ear system—were consistently associated with larger ABGs and more severe conductive hearing loss.

This finding corroborates previous international studies by Keefe (2012), Attias et al. (2022), and Aithal (2024), which determine that frequency-specific absorbance is a reliable objective predictor of the magnitude of conductive hearing loss (6–17). By demonstrating this correlation, our study represents the first investigation in Vietnam to show that WBT functions not only as a diagnostic modality but also as a prognostic indicator. The stiffening effect of the effusion directly impaired sound energy transmission, explaining the significant conductive deficit observed in the low-to-mid frequencies.

### 4.3 Clinical utility of combining WBT with kempthorne criteria

While WBT offers objective functional data, the Kempthorne criteria provide a standardized endoscopic assessment of tympanic membrane morphology. Our results suggest that combining these two methods can enrich clinical characterization beyond single-frequency tympanometry alone. While the 226-Hz tympanogram classifies middle-ear status mainly as type A/B/C, integration of WBT absorbance data with high-yield Kempthorne morphological features provides a more detailed profile of tympanic mobility and middle-ear mechanics, which may support clinical reasoning even when binary screening performance of 226-Hz testing is already high among surgically indicated ears.

The formal comparison of sensitivity, specificity, and AUC between standard care and the combined Kempthorne–WBT model (Table 3) must be interpreted in light of a diagnostic baseline paradox. Standard 226-Hz tympanometry achieved 100% sensitivity (31/31) in the surgically verified cohort, indicating that conventional testing identified every ear that ultimately required myringotomy. Accordingly, the manuscript does not claim that standard tympanometry fails to detect surgically indicated adult OME; rather, any incremental statistical advantage of the combined model in this study rests largely on specificity (and related NPV/AUC) differences derived from the surgically unverified reference-negative subset. These comparative estimates—particularly specificity 86.4%, NPV, and overall AUC—should therefore be interpreted with caution because non-operated ears were assumed disease-negative without myringotomy confirmation. Prospective blinding of WBT interpretation reduced interpretive bias but does not eliminate partial verification bias.

This combined approach was particularly valuable in adult patients, where OME can be chronic and insidious. The ability of WBT to detect subtle mechanical changes (Type E) even when conventional tympanometry was inconclusive allows for earlier intervention. Consequently, this protocol supported more timely decision-making regarding ventilation tube insertion, potentially preventing long-term complications such as adhesive otitis or atelectasis.

### 4.4 WBT absorbance and effusion viscosity

The high prevalence of glue effusion (87.1%) mirrors chronic adult OME in southern Vietnam. Although subgroup numbers for serous and mucoid effusion were small (*n* = 2 each), descriptive absorbance profiles (Table 4) suggested lower band absorbance in glue ears, supporting a mechanistic link between viscous effusion and middle-ear stiffness. This extends the morphological discussion beyond Type E curves alone.

#### 4.4.1 Sample size and statistical power

The *a priori* target of ≥28 surgically verified ears was met (*n* = 31). *Post hoc* calculations indicated adequate power (84.6%) to detect the observed AUC difference between standard care and the combined model. While the operative subset remains modest for viscosity-stratified analyses, the cohort was sufficiently powered for the prespecified primary diagnostic comparisons.

#### 4.4.2 Verification bias and reference-standard limitations

Only 31 of 66 ears underwent operative verification, introducing severe partial verification bias. Comparative analyses (Table 5) showed no significant differences in key preoperative variables between operated and non-operated ears, suggesting the verified subgroup was reasonably representative on measured covariates. Critically, however, the diagnostic accuracy analysis was based on a composite reference standard in which all non-operated ears were considered disease-negative. The absence of surgical verification in this group means that some ears classified as reference-negative may still have harbored occult or resolving middle-ear effusion. Such misclassification of true disease as reference-negative can artificially inflate or otherwise skew specificity (reported 86.4% for the combined model), NPV, and overall AUC estimates, depending on how those ears are labeled by each index test. Sensitivity estimates, by contrast, were derived solely from surgically confirmed fluid-positive ears and are comparatively robust to this mechanism—although they also reveal that standard 226-Hz tympanometry already detected all verified positives. We therefore emphasize that apparent advantages of the combined model on specificity, NPV, and AUC should not be overstated and require validation in cohorts with surgical verification of both disease-positive and disease-negative ears.

**Table 5:** Baseline and preoperative comparison between operated and Non-operated ears (*N*=66).

Characteristic/Variable	Operated Group (*n*=31)	Non-Operated Group (*n* = 35)	Test Statistic (*χ*²/t)	*p*-value
Age (years), Mea*n* ± SD	43.8 ± 16.9	45.9 ± 18.0	t = −0.48	0.633
Female gender, *n* (%)	17 (54.8%)	20 (57.1%)	χ² = 0.04	0.852
Duration of symptoms ≥ 12 weeks, *n* (%)	11 (35.5%)	11 (31.4%)	χ² = 0.12	0.725
Tinnitus, *n* (%)	23 (74.2%)	26 (74.3%)	χ² < 0.01	0.993
Hearing loss, *n* (%)	21 (67.7%)	22 (62.9%)	χ² = 0.17	0.680
Air-fluid level present, *n* (%)	26 (83.9%)	29 (82.9%)	χ² = 0.01	0.913
Absent TM mobility, *n* (%)	24 (77.4%)	25 (71.4%)	χ² = 0.30	0.584
Yellowish TM discoloration, *n* (%)	25 (80.6%)	28 (80.0%)	χ² = 0.00	0.949
PTA Hearing Threshold (dB HL), Mean ± SD	33.1 ± 11.8	31.8 ± 10.7	t = 0.47	0.640
226-Hz Tympanogram Type B (Flat), *n* (%)	22 (71.0%)	23 (65.7%)	χ² = 0.22	0.638
Peak Pressure (daPa), Mean ± SD	−178.5 ± 72.1	−170.1 ± 68.7	t = −0.48	0.630
WBT Type E Curve (Reverse V shape), *n* (%)	24 (77.4%)	26 (74.3%)	χ² = 0.09	0.768
WBT Low-Freq Absorbance (250–2,000 Hz), Mean %	10.8 ± 5.1%	11.5 ± 5.7%	t = −0.52	0.605

SD, standard deviation; TM, tympanic membrane; PTA, pure-tone average; WBT, wideband tympanometry.

All *p*-values > 0.05 confirm no statistically significant difference between operated and non-operated ears across core parameters.

#### 4.4.3 Separation of eligibility screening from index-test evaluation

A central methodological concern is potential circularity if the Kempthorne >5 enrollment threshold were reused within the evaluated diagnostic model. We addressed this by (1) prespecifying that >5 served only as an eligibility criterion; (2) defining the combined model using independent high-yield morphological features (air-fluid level and absent mobility) rather than the binary >5 threshold; and (3) conducting a sensitivity analysis with cohort-median Kempthorne score plus WBT, which reproduced similar accuracy.

#### 4.4.4 Interpretation of sensitivity, specificity, and the diagnostic baseline paradox

Because all 31 operated ears had confirmed fluid, sensitivity was computed from true-positive index-test classifications among operated ears, whereas specificity required the 35 non-operated ears classified as reference-negative. The observation of 100% sensitivity with sub-100% specificity for standard tympanometry reflects abnormal 226-Hz tracings (type B or C) in all fluid-confirmed ears coupled with type A tracings in a subset of clinically stable, non-operated ears. This pattern creates an interpretive paradox relative to literature narratives that emphasize limited utility of single-frequency tympanometry: in the present surgically enriched positive set, conventional tympanometry was a perfect detector of operative fluid, so the added value argued for WBT/Kempthorne combination concerns complementary morphological information and putative specificity gains—not rescue of missed surgical cases. Although PPV and NPV are reported alongside sensitivity and specificity to aid clinical interpretation, these predictive values—and overall diagnostic accuracy/AUC—remain vulnerable to misclassification of non-operated ears under the composite reference standard and should not be regarded as definitive estimates of real-world performance.

### 4.5 Limitations

This single-center prospective study has some limitations. First, convenience sampling at one tertiary hospital may limit generalizability. Second, and most importantly for the accuracy metrics presented here, the composite reference standard classified all non-operated ears as disease-negative without surgical confirmation; residual occult or resolving effusion in this group may therefore inflate or skew specificity (including the combined-model estimate of 86.4%), PPV, NPV, and overall AUC through severe partial verification bias. Third, because standard 226-Hz tympanometry achieved 100% sensitivity among surgically verified ears, the incremental advantage attributed to the combined model is confined largely to specificity contrasts in the unverified control subset and should not be interpreted as superior detection of operative-level disease. Fourth, no longitudinal follow-up was performed after ventilation tube insertion. Fifth, comparison with Aithal et al. (2020) used published aggregate means rather than individual-level pediatric data. Finally, non-glue effusion subgroups were too small for robust inferential testing.

## 5 Conclusion

This study showed that Wideband Tympanometry (WBT) provides a detailed functional assessment of the middle ear in adults with OME and, when combined with specific Kempthorne morphological features, yields complementary clinical information beyond single-frequency classification alone. Within a composite reference standard with severe partial surgical verification, formal comparison suggested higher specificity and AUC for the combined Kempthorne-feature + WBT model than for standard 226-Hz tympanometry (Table 3). However, standard 226-Hz tympanometry achieved 100% sensitivity in the surgically verified cohort; therefore, the combined model’s apparent advantage does not reflect improved detection of myringotomy-confirmed effusion and rests largely on specificity, NPV, and AUC estimates derived from non-operated ears assumed to be disease-negative. Specificity, PPV, NPV, and overall diagnostic accuracy must be interpreted cautiously under this partial verification framework. These findings should be viewed as hypothesis-generating rather than definitive evidence of superiority of the combined model and warrant confirmation in cohorts with more complete surgical verification of both disease-positive and disease-negative ears.

## Data Availability

The original contributions presented in the study are included in the article/Supplementary Material, further inquiries can be directed to the corresponding author.

## Appendix. Kempthorne scoring table.

**APPENDIX 1:** Complete kempthorne criteria referenced in the materials and methods section [adapted from kempthorne and giebink, 1991 (11)].

Feature	Finding	Score
Opacity/Transparency	Normal	0
	Partial opacity	1
	Complete opacity	2
Color	White-gray	0
	Partial yellow	1
	Complete yellow	2
Air-fluid level/Bubbles	Absent	0
	Present	8
Position/Retraction	Normal	0
	Retracted	1
	Flat	2
	Partial tension effusion	4
	Full effusion	6
	Bulging	8
Mobility	Normal	0
	Reduced	2
	Absent	4
Light reflex	Normal	0
	Reduced or absent	1

Interpretation: 0–2 points = no effusion; 3–5 points = possible effusion; >5 points = effusion present (inclusion threshold in this study).

## References

[1] Mills R Hathorn I. Aetiology and pathology of otitis media with effusion in adult life. J Laryngol Otol. (2016) 130(5):418–24. 10.1017/S0022215116000943 26976514

[2] Tuomi L Fransson P Wennerberg J Finizia C. Otitis media with effusion in Africa: prevalence and associated factors—a systematic review and meta-analysis. Laryngoscope Investig Otolaryngol. (2020) 5(6):1205–16. 10.1002/lio2.490 33364413 PMC7752044

[3] Nguyen TV Nguyen TH Ho AK Nguyen TNL. Bacterial culture results and antibiograms in chronic rhinosinusitis patients in Mekong Delta, Vietnam. Open Microbiol J. (2024) 18:e18742858322304. 10.2174/0118742858322304240819110420

[4] Stuppert L Nospes S Bohnert A Läßig AK Limberger A Rader T. Clinical benefit of wideband-tympanometry: a pediatric audiology clinical study. Eur Arch Otorhinolaryngol. (2019) 276(9):2433–9. 10.1007/s00405-019-05498-2 31175454

[5] Parlea E Georgescu M Calarasu R. Tympanometry as a predictor factor in the evolution of otitis media with effusion. J Med Life. (2012) 5(4):452–4. PMID: 23483082 23346249 PMC3539835

[6] Aithal S Venkatesh A Kei J. Wideband absorbance predicts the severity of conductive hearing loss in children with otitis media with effusion. Int J Pediatr Otorhinolaryngol. (2024) 180:111703. 10.1016/j.ijporl.2024.111703 38082489

[7] Karuppannan A Barman A Mamatha NM. Wideband absorbance pattern and its diagnostic value in adults with middle ear effusions and tympanic membrane perforation. J Int Adv Otol. (2024) 20(2):158–63. 10.5152/iao.2024.231048 39145608 PMC11114253

[8] Karuppannan A Barman A. Evaluation of wideband absorbance tympanometry in adults with abnormal positive and negative middle ear pressure. J Hear Sci. (2020) 10(4):40–7. 10.17430/JHS.2020.10.4.5

[9] Attias J Shahnaz N Efrat C Westerberg B Lea J David E. Wideband tympanometry in adults with severe to profound hearing loss with and without cochlear implants. Appl Sci. (2022) 12(17):8879. 10.3390/app12178879

[10] Aithal V Aithal S Kei J Driscoll C. Wideband absorbance in ears with otitis media with effusion: a comparative study. J Am Acad Audiol. (2020) 31(3):197–205.

[11] Kempthorne J Giebink GS. Pediatric approach to the diagnosis and management of otitis media. Otolaryngol Clin North Am. (1991) 24(4):905–27. 10.1016/S0030-6665(20)31099-9 1870882

[12] Galić MZ Klančnik M. Adenoid size in children with otitis media with effusion. Acta Clin Croat. (2022) 61(3):532–9. 10.20471/acc.2022.61.03.20 PMC8907955 35282481

[13] Qureishi A Lee Y Belfield K Birchall JP Daniel M. Update on otitis media—prevention and treatment. Infect Drug Resist. (2014) 7:15–24. 10.2147/IDR.S39637 24453496 PMC3894142

[14] Şahin MI Özyürek DD Vural A Zararsız G Ketenci I Ünlü Y. Can wideband tympanometry predict the prognosis of Otitis media with effusion? J Audiol Otol. (2025) 29(2):95–102. 10.7874/jao.2021.00633 PMC12046198 35538866

[15] Keskin Yıldız M Atay G Mergen EK Aksoy S Böke B. A novel approach to the follow up of children with otitis media with effusion: wideband absorbance findings. Eur Arch Otorhinolaryngol. (2025) 282(9):4487–96. 10.1007/s00405-025-09355-3 PMC12423127 40220181

[16] National Institute for Health and Care Excellence. Surgical Management of otitis Media with Effusion in Children. NICE Clinical Guideline CG60. London: National Institute for Health and Care Excellence (NICE); (2008).

[17] Keefe DH Sanford CA Ellison JC Fitzpatrick DF Gorga MP. Wideband aural acoustic absorbance predicts conductive hearing loss in children. Int J Audiol. (2012) 51(12):880–91. 10.3109/14992027.2012.721935 23072655 PMC3693460

